# Bilateral Transient Osteoporosis of the Hip in Pregnancy: Diagnostic Challenges, MRI-Based Approach, and Multidisciplinary Management

**DOI:** 10.3390/diseases14060208

**Published:** 2026-06-10

**Authors:** Pavol Zubor, Kristen Olav Lind, Jozef Visnovsky, Petra Zuborova, Guri Grimnes, Cato Kjærvik

**Affiliations:** 1 Department of Obstetrics and Gynaecology, Nordland Hospital Trust, Vesterålen Hospital, 8450 Stokmarknes, Norway; 2 OBGY Health & Care Ltd., 01001 Zilina, Slovakia; zuborovap@gmail.com; 3 Faculty of Health Care, Catholic University, 03401 Ruzomberok, Slovakia; jozo.visnovsky@gmail.com; 4 VISNOVSKI Ltd., 03601 Martin, Slovakia; 5 Division of Internal Medicine, University Hospital of North Norway, 9037 Tromsø, Norway; guri.grimnes@unn.no; 6 Department of Clinical Medicine, UiT—The Arctic University of Norway, 9019 Tromsø, Norway; catokjarvik@gmail.com; 7 Department of Surgery, Orthopaedic Section, Nordland Hospital Trust, Vesterålen Hospital, 8450 Stokmarknes, Norway

**Keywords:** transient osteoporosis of the hip, pregnancy, bilateral, bone marrow oedema, MRI, DXA, multidisciplinary management

## Abstract

Background: Pregnancy-related transient osteoporosis of the hip (PR-TOH) is an uncommon and frequently underdiagnosed condition that typically presents with acute hip pain during late pregnancy or the early postpartum period. Because its clinical presentation is nonspecific and overlaps with pregnancy-related pelvic girdle pain, the diagnosis is often delayed, and the initial management is suboptimal. Although bilateral involvement has been reported, comparative data on diagnostic work-up, multidisciplinary management, and follow-up remain limited. Case Presentation: We report a case of bilateral PR-TOH in a 35-year-old Caucasian primigravida (G1, P0) who presented at 31 + 6 weeks of gestation with progressively worsening bilateral hip pain that culminated in severe functional impairment and wheelchair dependence. Initial ultrasound, laboratory work-up, and rheumatological screening were inconclusive, and intra-articular corticosteroid injections failed to relieve symptoms and were temporally associated with deterioration of glycaemic control and a periorbital and palmar eczematous rash. Magnetic resonance imaging (MRI) demonstrated diffuse bone marrow oedema in both femoral heads with preserved articular cartilage and no evidence of avascular necrosis, supporting a diagnosis of bilateral PR-TOH. Postpartum dual-energy X-ray absorptiometry (DXA) confirmed reduced bone mineral density at both femoral necks (Z-scores below −2.0). Pregnancy was prolonged until 37 + 4 weeks, and delivery was by elective caesarean section. Postpartum care included analgesia, calcium and vitamin D supplementation, structured physiotherapy, and a graded weight-bearing rehabilitation programme. Bone mineral density improved markedly on follow-up DXA at six months, with complete clinical recovery and no further imaging abnormalities at 12, 24, and 30 months. Conclusions: PR-TOH should be considered in pregnant or postpartum women with persistent hip pain and progressive functional limitation. MRI is the key imaging modality for early diagnosis and for excluding alternative causes, whereas DXA remains the reference standard for quantifying bone mineral density and monitoring recovery. Bilateral presentations require a multidisciplinary, individualised approach that addresses both maternal and obstetric outcomes.

## 1. Introduction

Transient osteoporosis of the hip (TOH) is an uncommon, self-limiting disorder that is most often described in middle-aged men and in women during late pregnancy or the early postpartum period [1,2,3,4,5]. Clinically, it is characterised by progressive, atraumatic hip pain accompanied by transient demineralisation of the proximal femur on imaging, without joint-space narrowing or articular destruction, and in the absence of identifiable causes of synovitis or secondary osteoporosis.

The condition was first described by Curtis and Kincaid in 1959 in a series of three pregnant women [6]. Symptoms typically begin with insidious, atraumatic, dull pain in one or both hips that gradually worsens with weight bearing and may progress to severe restriction of mobility [7]. The hip is the most frequently affected site (approximately three-quarters of cases) [8], but isolated or migratory involvement of the knee, ankle, or foot has also been reported during the same pregnancy [9,10]. TOH is biologically and clinically a self-limiting entity in which pain and the radiological abnormalities usually resolve spontaneously within 6–8 months after delivery [7,8]. Atraumatic femoral neck fracture has been reported in up to 12% of cases [11]; outcomes after surgical fixation, with or without antiresorptive therapy, are generally favourable [12,13,14].

Although several recent reviews and case series have improved awareness of PR-TOH [8,15,16,17], bilateral involvement during pregnancy remains uncommon, and the published literature still consists predominantly of single-patient reports with heterogeneous diagnostic workups and management strategies. Specifically, only a limited number of bilateral PR-TOH cases diagnosed in the third trimester have been documented with complete obstetric, imaging, endocrinological, and orthopaedic follow-up extending beyond two years. The present report adds to that body of evidence by describing in detail (i) the diagnostic pathway from non-specific musculoskeletal complaints to MRI-based confirmation, (ii) the practical consequences of intra-articular corticosteroid administration in a patient with concurrent gestational diabetes, (iii) a structured non-pharmacological postpartum rehabilitation programme, and (iv) the longitudinal evolution of bone mineral density assessed by DXA up to 30 months after delivery.

Given the rarity of bilateral PR-TOH and the absence of formal management guidelines, this stepwise description is intended as a practical reference for clinicians who may encounter this entity during pregnancy.

## 2. Case Presentation

The patient was diagnosed and managed at the Department of Obstetrics and Gynecology and the Department of Orthopaedic Surgery, Nordland Hospital Trust, Vesterålen Hospital (Stokmarknes, Norway), with subsequent endocrinological follow-up at the University Hospital of North Norway, Tromsø. The case occurred between September 2023 and the final follow-up examination was completed in March 2026.

A 35-year-old Caucasian woman (G1, P0) was referred to the emergency department at 36 + 4 weeks of gestation (wg) because of gradually worsening bilateral hip pain and a recurring sensation that her legs were ‘giving way’. Obstetric examination of both mother and foetus revealed no concerning features. The pregnancy had been complicated by insulin-treated gestational diabetes, and foetal growth was within the 12th percentile, with normal cardiotocography and ultrasound findings.

Baseline anthropometric and clinical characteristics were as follows: height 166 cm, pre-gravid weight 53 kg, pre-gravid body mass index (BMI) 19.2 kg/m^2^, total gestational weight gain [+10 kg]. She was a non-smoker, denied alcohol use, and reported a balanced mixed diet with no history of restrictive eating. She had taken a standard prenatal multivitamin from an early pregnancy that contained 400 IU of vitamin D and 200 mg of calcium per day; no additional calcium or vitamin D supplementation had been prescribed before symptom onset. She had no personal or family history of fragility fracture, osteoporosis, or endocrine disorders.

Her gynaecological history included long-standing infertility related to diminished ovarian reserve (anti-Müllerian hormone 3.5 pmol/L), which had required in vitro fertilisation and embryo transfer (IVF-ET) of a single blastocyst. She had no prior gynaecological surgery, no known allergies, and no significant medical history before the pregnancy. Gestational diabetes had been diagnosed at 27 + 5 wg and was managed with metformin and insulin. At 31 + 6 wg she reported sudden-onset left hip pain that worsened progressively and required oral analgesics and crutches, as well as zopiclone 5 mg at bedtime for pain-related insomnia. She reported no preceding trauma and no previous back or hip disorders and was afebrile on initial presentation. Laboratory investigations showed no evidence of infection (C-reactive protein <4 mg/L; leucocytes 14.1 × 10^9^/L).

At 32 + 3 wg she was referred to the orthopaedic department because she was unable to mobilise or bear weight on the left leg. Hip ultrasonography (Figure 1) showed a small intra-articular fluid collection of 3–4 mm at the femoral neck with diffuse capsular thickening, interpreted as left coxitis. She was treated with oral analgesia (paracetamol and paracetamol–codeine) and referred to a rheumatologist, who excluded an infectious aetiology (CRP <4 mg/L; leucocytes 14.1 × 10^9^/L). Rheumatological screening was negative on both initial work-up and at 6 weeks postpartum and 12 months after delivery (negative ANA, anti-CCP IgG 0.8 U/mL [reference <5] and rheumatoid factor <3.5 IU/mL; negative multiplex flow immunopanel comprising dsDNA, Chromatin IgG, Ribosomal P protein IgG, Sm IgG, SSA-Ro52 IgG, SSA-Ro60 IgG, SSB IgG, RNP IgG, SmRNP, Centromer B IgG, Scl-70 IgG, Jo-1 IgG, and CCP IgG).

Because of the persistent disabling pain and the absence of an alternative diagnosis, ultrasound-guided intra-articular injections (0.7 mL of 1% lidocaine and 0.6 mL of triamcinolone acetonide 40 mg/mL [Kenacort]) were administered into both hip joints after symptoms developed on the right side at 34 + 2 wg. Figure 2 shows the bilateral synovial fluid collections before ultrasound-guided arthrocentesis; aspirate analysis was negative for bacterial or viral infection, contained no crystals, but showed an elevated synovial leucocyte count (2.2 × 10^9^/L; reference <0.2). At 34 + 5 wg she required a wheelchair because of new bilateral knee pain that prevented walking even with crutches.

Shortly after the bilateral intra-articular injections, two adverse events were observed:

(i) A progressive deterioration of glycaemic control that required stepwise increases in insulin dose, consistent with the known hyperglycaemic effect of systemic absorption of intra-articular corticosteroids in patients with insulin-dependent gestational diabetes, and
(ii) An itchy erythematous rash with periorbital swelling and nummular eczematous plaques on the hands. Although a coincidental dermatological flare could not be ruled out, the close temporal relationship suggests a probable hypersensitivity reaction to either the local anaesthetic or the corticosteroid vehicle. Both events were discussed in the multidisciplinary management and are addressed further in the Discussion.

Despite intra-articular corticosteroid therapy, hip pain did not improve, and the patient was urgently re-admitted one week later. In addition to the rash described above, she was completely immobile, severely sleep deprived, and showed marked psychological distress in the context of intractable pain and progressive loss of autonomy. She was hospitalised at 36 + 2 wg and treated with opioid analgesia, topical corticosteroids (mometasone cream, aluminium acetotartrate dressings), and oral cetirizine, with topical ophthalmic treatment for the periorbital eruption. Insulin requirements continued to rise, and foetal ultrasonography demonstrated a small-for-gestational-age foetus (10th–12th percentile) in breech presentation. A planned caesarean section was performed at 37 + 4 wg, delivering a healthy female newborn weighing 3265 g (Apgar 9–10–10).

A multidisciplinary case conference raised a strong suspicion of bilateral PR-TOH, and dedicated investigations were performed in the early postpartum period. Pelvic and hip radiographs showed bilateral diffuse osteopenia of the femoral heads without other abnormalities (Figure 3, R: right hip; L: left hip, presented as two separate radiographs). MRI demonstrated findings compatible with bilateral PR-TOH (Figure 4). Serum bone-specific alkaline phosphatase was elevated (28 µg/L; reference 5.5–25), indicating increased bone turnover. Additional bone turnover markers, including serum C-terminal telopeptide of type I collagen (CTX) and procollagen type I N-terminal propeptide (P1NP), were not measured at the time of diagnosis.

Postpartum care was uncomplicated. The patient was advised to discontinue breastfeeding to limit further bone resorption. Conservative management was continued with oral analgesia (diclofenac 50 mg and paracetamol 1 g as needed), calcium carbonate 1500 mg with cholecalciferol 2400 IU per day (Calcigran Forte, three tablets daily), a single oral loading dose of 100,000 IU vitamin D, and a daily multivitamin supplement. Treatment with a bisphosphonate was discussed but not initiated because of the limited safety and efficacy data in lactation-eligible women, the absence of fragility fracture, and the expected spontaneous recovery of bone mineral density (see Discussion). Parathyroid hormone analogues (teriparatide) and denosumab were also considered but not prescribed for the same reasons; in addition, the Norwegian national reimbursement scheme does not cover teriparatide in women younger than 40 years without a documented fragility fracture. Bone mineral density was assessed by DXA, and the patient was referred for endocrinological evaluation. Ultrasound on postpartum day 10 showed regression of the right hip effusion to 5.4 mm (total joint diameter 9.4 mm), and no residual collection in the left hip (capsular thickness 6.9 mm).

Bone mineral density was assessed on postpartum day 10 using a Hologic Discovery Wi densitometer (Hologic Inc., Marlborough, MA, USA) with daily phantom calibration. In accordance with the position of the International Society for Clinical Densitometry, results in this premenopausal patient are reported as age-, sex-, and ethnicity-matched Z-scores rather than T-scores. The left femoral neck Z-score was −2.7, and the right femoral neck Z-score was −2.9 (Figure 5), both below the ‘expected range for age’ threshold of −2.0. The lumbar spine (L1–L4) Z-score was −1.1 (slightly reduced). Serum thyroid-stimulating hormone, free T4, 25-hydroxyvitamin D (127 nmol/L), total calcium (2.34 mmol/L), ionised calcium (1.22 mmol/L), phosphate (1.34 mmol/L), and parathyroid hormone (4.9 pmol/L) were all within the normal range.

The structured rehabilitation programme was initiated on postpartum day 14 and was supervised by an experienced physiotherapist with weekly clinical reassessment by the treating orthopaedic surgeon. Two supervised sessions per week of approximately 60 min were combined with a daily home programme. Phase 1 (weeks 0–4) focused on protected weight bearing (two crutches, approximately 30% body-weight loading), isometric quadriceps and gluteal activation, gentle hip range-of-motion exercises within a pain-free arc, and lumbo-pelvic stabilisation. Phase 2 (weeks 5–10) introduced progressive partial weight-bearing, closed-chain strengthening (mini-squats, hip abduction, and bridging), and stationary cycling. Phase 3 (from week 11) comprised full weight-bearing with crutch weaning, balance and proprioception training, low-impact aerobic activity (cycling, aquatic exercise, and Nordic walking), and progressive resistance training of the hip and pelvic musculature. High-impact loading and contact sports were avoided for the first 12 months. The patient adhered well to the programme and reported a steady, parallel improvement in pain and function.

At the seven-week postpartum review, the patient was able to stand upright with minimal hip pain and walked short distances with the support of crutches.

DXA repeated six months after delivery showed substantial improvement in bone mineral density at the femoral neck (left Z-score −1.3, +29.3%; right Z-score −1.3, +34.1%) without significant change at the lumbar spine (Figure 6). The patient was advised to continue annual follow-up until complete normalisation of femoral neck bone density. At 12, 24, and 30 months, she remained asymptomatic and free of imaging abnormality, with normal serum calcium, phosphate, parathyroid hormone, and vitamin D.

## 3. Discussion

Transient osteoporosis of the hip is a self-limiting condition of unknown aetiology that usually resolves with conservative treatment, although it can be complicated by atraumatic fracture or, less often, evolve towards avascular necrosis. Although TOH appears slightly more frequent in men overall, in women, it almost always occurs in the third trimester or the early postpartum period. In contrast to avascular necrosis, PR-TOH typically resolves within 6–8 months after delivery [7], with MRI signal abnormalities normalising over 3–6 months [8,18]. Recovery is closely linked to the return of menstrual function, the cessation of lactation, and the homeostatic mechanisms that protect the maternal skeleton during exclusive breastfeeding [19]. After weaning, bone mineral density increases by approximately 0.5–2% per month and usually returns to pre-pregnancy values within 12 months [20].

Recent literature has refined the clinical picture of PR-TOH. The systematic review by Galanis et al. [8] of 345 PR-TOH-linked articles highlighted the predominance of third-trimester onset, the high diagnostic value of MRI, and the generally good prognosis under conservative management. The case series of Toussia-Cohen et al. [15] of 34 women confirmed older maternal age, frequent use of assisted reproduction technologies, and a high prevalence of smoking and family history of osteoporosis as recurrent features. Quaresima et al. [16] proposed PR-TOH as a non-obstetric indication for elective caesarean section, an approach that mirrors the management of the present case. Morton and Savard-Heppel [17] recently reviewed contemporary evidence and emphasised MRI as the diagnostic modality of choice and the need to weigh pharmacological options on an individual basis. Compared with these publications, the bilateral involvement, the simultaneous occurrence of iatrogenic complications of intra-articular corticosteroid administration, the structured non-pharmacological rehabilitation programme and the longitudinal DXA-based follow-up beyond 24 months distinguish the present report.

The aetiology of TOH is still incompletely understood and is best regarded as multifactorial. Curtis and colleagues [6] proposed mechanical compression of the obturator nerve and reduced arterial perfusion of the joint. Others have suggested local compression of pelvic vessels and nerves by the gravid uterus [21], hormonal factors [22], reflex sympathetic dystrophy of the Sudeck type [23,24], venous stasis [25], transient synovitis [26], or atraumatic subclinical osteonecrosis [27]. None of these single hypotheses explains the occurrence of TOH in men [2,28] or in non-pregnant or young women [29,30,31], and current opinion favours a multifactorial mechanism in which physical, vascular, and hormonal factors interact [32].

Maliha et al. [33] linked PR-TOH to obturator nerve compression, vascular obstruction by the foetus, pregnancy-related impairment of fibrinolysis with secondary bone ischaemia and vitamin D deficiency. Because PR-TOH has also been documented in the first trimester, purely mechanical theories are unlikely to be sufficient. Emami et al. [34] supported a microvascular mechanism leading to focal bone ischaemia. In a case–control study, Hadji et al. [11] identified immobility, dental disease, and limited exercise during childhood as associated factors, consistent with a multicausal pathogenesis. More recent work has highlighted the role of low peak bone mass, accelerated pre-conception bone resorption, and monogenic bone disorders (notably pathogenic variants in LRP5, WNT1, COL1A1, and COL1A2) as predisposing factors that may become clinically apparent under the metabolic load of pregnancy [35,36]. Other recognised risk factors include chronic inflammatory disease, previous trauma, vascular disorders, neoplasia, alcohol intake, smoking, glucocorticoid use, and hypothyroidism [8,37]. Advanced maternal age, low BMI, family history of osteoporosis, and conception by IVF are also considered potential contributors. Toussia-Cohen et al. [15] reported a mean maternal age of 34.2 years, a positive family history in 29.4%, smoking in 47.1%, and IVF conception in 32.4%; the patient in the present report shares several of these features (advanced maternal age, low pre-gravid BMI, and IVF conception).

Distinguishing PR-TOH from physiological pregnancy- and lactation-associated bone loss (PAO/LAO) is difficult, because the latter is itself driven by hypooestrogenaemia, prolactin-mediated bone resorption and the marked increase in foetal calcium demand during the third trimester, with subsequent spontaneous recovery [38].

Musculoskeletal pain at the hip, lower back, symphysis, sacroiliac joints, or groin is one of the most frequent complaints during pregnancy. It is commonly attributed to increased body weight, exaggerated lumbar lordosis with axial loading, and to the joint-laxity effect of relaxin and other gestational hormones [39]. In a busy clinical setting, these symptoms are easily interpreted as benign pelvic–girdle pain, which may delay the diagnosis of PR-TOH, particularly among clinicians who have little exposure to this condition or to PAO/LAO.

Plain radiography is usually the first imaging examination, but it can be normal in the first weeks because radiographic osteopenia takes 4–8 weeks to appear after the onset of pain. This often delays the diagnosis, particularly where MRI is not readily available, and patients are initially managed with non-steroidal anti-inflammatory drugs and crutches. By contrast, MRI can detect bone marrow oedema as early as 48 h after symptom onset [40].

MRI is the most sensitive imaging modality for the early recognition of PR-TOH. The hallmark pattern is a homogeneous bone marrow oedema involving the femoral head with or without neck extension, showing low T1 and high T2 (and STIR) signal, preserved articular cartilage, and absence of subchondral changes [41]. These features help differentiate PR-TOH from avascular necrosis, which can have a strikingly similar early appearance [42] but carries a far worse prognosis and may also occur during pregnancy [43]. Interpretation of these images should be performed by a radiologist familiar with peripartum musculoskeletal disease. It is important to emphasise that bone marrow oedema on MRI is a non-specific imaging sign and is not synonymous with a reduction in bone mineral density. MRI is therefore best understood as the gold standard for early detection of the characteristic oedema pattern and for ruling out alternative pathology (stress and fragility fracture, avascular necrosis, septic and inflammatory arthritis, soft-tissue or bone neoplasia), while DXA remains the reference method for quantifying bone mineral density and for confirming the underlying osteoporosis after delivery.

DXA played a central role in the present case. According to the position of the International Society for Clinical Densitometry, the WHO T-score-based definition of osteoporosis (≤−2.5) applies to postmenopausal women and to men aged 50 years or older, but not to premenopausal women. In premenopausal women, results should be reported as Z-scores, and a Z-score of −2.0 or lower is described as ‘below the expected range for age’. A diagnosis of osteoporosis in this population requires a low Z-score in combination with a documented secondary cause or with a clinically relevant fragility fracture. In the present case, femoral neck Z-scores of −2.7 and −2.9 in the context of a clinically and radiologically confirmed PR-TOH satisfy this combined criterion. The figure legends and the case description have been corrected accordingly so that all DXA data are now reported as Z-scores, with T-scores removed from the main text.

The principal differential diagnoses of acute hip pain in late pregnancy include avascular necrosis of the femoral head, sacral or femoral neck stress fracture, septic arthritis, inflammatory arthritis (e.g., early spondyloarthritis or rheumatoid disease), pelvic–girdle pain, and, more rarely, bone or soft-tissue neoplasia. MRI features that favour PR-TOH include diffuse oedema confined to the femoral head and neck, an intact subchondral plate, preserved articular cartilage, and absence of the double-line sign of avascular necrosis, of fracture lines (linear hypointense bands on T1), and of focal mass lesions. The combination of these features with normal inflammatory markers, sterile arthrocentesis, and a negative rheumatological screen, as in the present case, supports the diagnosis.

Both ultrasound examinations in this patient (Figure 1 and Figure 2) demonstrated a small intra-articular fluid collection with capsular oedema. In PR-TOH, a small reactive synovitis is a recognised but inconstant finding and probably reflects the secondary irritation of the synovial membrane induced by the underlying subchondral oedema rather than a primary inflammatory process. Larger or recurrent effusions should prompt sampling to exclude septic or crystal-induced arthritis, as was performed in the present case.

The bilateral intra-articular triamcinolone injections administered before diagnosis deserve specific comment. In insulin-dependent gestational diabetes, systemic absorption of intra-articular corticosteroid is well known to disrupt glycaemic control and to require substantial transient increases in insulin dose, as observed here. The simultaneous appearance of an itchy periorbital and palmar eczematous reaction further raises the possibility of hypersensitivity to either the local anaesthetic vehicle or to the preservatives in the corticosteroid preparation. These observations support a more cautious indication for intra-articular corticosteroids in pregnant women with insulin-dependent diabetes and unexplained hip pain, and underline the need for an early diagnostic MRI before therapeutic infiltration.

Wilson et al. [44] were among the first to describe, in 1988, the typical T1- and T2-weighted MRI appearance of TOH in a series of 10 patients with hyperaemia, oedema and increased bone turnover, coining the term ‘transient bone marrow oedema syndrome’; this was confirmed shortly afterward by Bloem [45] in three additional cases. In the present case, the resolution of the high T2 signal paralleled the gradual relief of pain, consistent with these early reports.

Because PR-TOH has a generally favourable prognosis and complete recovery of bone mineral density is the rule, the cornerstone of treatment is joint protection (limited weight bearing, use of crutches or a wheelchair during pregnancy, labour, and the early postpartum period), oral analgesia (paracetamol or NSAIDs, the latter avoided in the third trimester), and calcium and vitamin D supplementation. The aim is to prevent microfractures, control pain, and maintain functional independence.

Because severe demineralisation can occasionally lead to femoral neck fracture and the need for surgical fixation, several authors have explored pharmacological strategies aimed at shortening recovery time. Samdani et al. [7] were the first to report successful pain control with an antiresorptive agent (alendronate) in PR-TOH, suggesting a possible role for bisphosphonates in reducing fracture risk and accelerating return to function. Spinarelli et al. [12] subsequently described complete functional recovery within three months in pregnant women with TOH-related fractures treated with alendronic acid 70 mg/week or risedronate 35 mg/week combined with vitamin D [46]. The decision not to administer bisphosphonates, denosumab, or teriparatide to the present patient was based on the following considerations:

(i) absence of fragility fractures;
(ii) the high probability of spontaneous bone density recovery after delivery and cessation of lactation, supported by our longitudinal DXA data;
(iii) the limited safety information on bisphosphonate transfer to breast milk and on long-term skeletal retention in young women of reproductive age;
(iv) the lack of formal indication for teriparatide in this clinical context and its non-reimbursement in Norway for women younger than 40 years without fragility fracture; and
(v) the absence of robust evidence from meta-analyses on antiresorptive therapy (bisphosphonates, calcitonin, denosumab, romosozumab) in TOH has not confirmed the favourable effects suggested by individual case reports [47,48].

Recent data on teriparatide in pregnancy- and lactation-associated osteoporosis are encouraging [49], but combined supplementation with calcium and vitamin D, together with joint protection and structured rehabilitation, remains the most widely recommended approach [50]. Our patient followed this strategy, with additional vitamin K supplementation and regular physiotherapy, and showed a very favourable response.

Because data on the safety and efficacy of bisphosphonates in PR-TOH are derived almost exclusively from case reports, and meta-analyses have not shown a clear benefit of antiresorptive treatment, pharmacological therapy is not part of standard recommendations for this condition.

A limitation of this report is the absence of a more comprehensive bone turnover panel, including serum CTX and P1NP measurements. Only bone-specific alkaline phosphatase was assessed, which alone is insufficient for a complete evaluation of bone remodelling activity. Nevertheless, the diagnosis of bilateral PR-TOH in our patient was supported by the characteristic clinical course, MRI findings, and postpartum DXA-confirmed reduction in femoral neck bone mineral density. Future reports should ideally include both bone formation and bone resorption markers to allow a more detailed metabolic characterization of PR-TOH during pregnancy and postpartum.

In summary, PR-TOH is an uncommon but clinically important condition that is easily overlooked because of its non-specific presentation and overlap with common musculoskeletal complaints in pregnancy. The present case underlines the importance of a high index of suspicion in women with persistent or progressive hip pain and functional impairment, the central role of early MRI in establishing the diagnosis and excluding alternative pathology, the reference role of DXA in quantifying bone mineral density and in monitoring recovery, and the value of a multidisciplinary, individualised management plan that integrates obstetrics, orthopaedics, endocrinology, and rehabilitation.

## 4. Conclusions

PR-TOH is an uncommon and frequently underdiagnosed condition that can substantially impair maternal well-being and pregnancy outcomes. Early recognition in women presenting with acute hip or back pain and progressive functional limitation requires a high index of suspicion and a multidisciplinary approach. MRI is the imaging modality of choice for early diagnosis and for excluding alternative causes of pregnancy-related hip pain. DXA remains the reference standard for quantifying bone mineral density and monitoring recovery and should be performed in the early postpartum period and at follow-up. Bone turnover markers may provide complementary information. Greater clinical awareness of PR-TOH should translate into earlier diagnosis, more individualised management, and better maternal and obstetric outcomes.

## Figures and Tables

**Figure 1:**
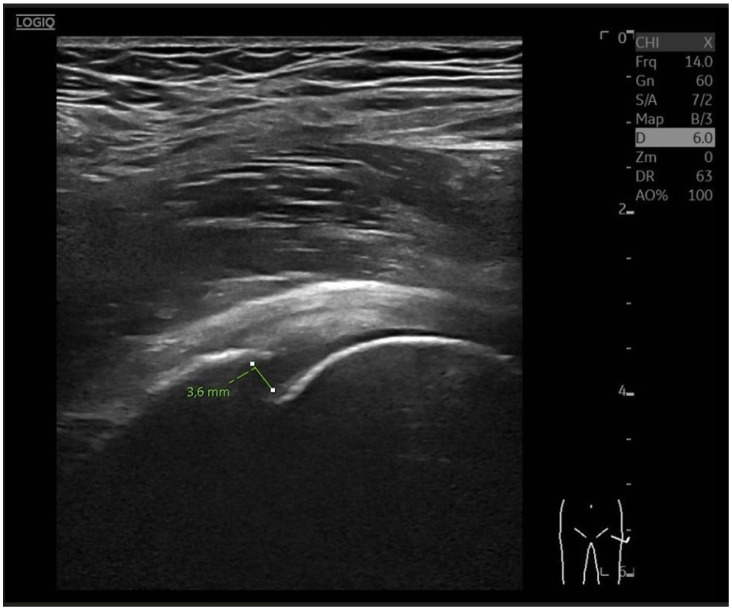
Ultrasound scan of the left hip joint showing a discrete fluid collection of 3–4 mm at the femoral neck and moderate capsular oedema.

**Figure 2:**
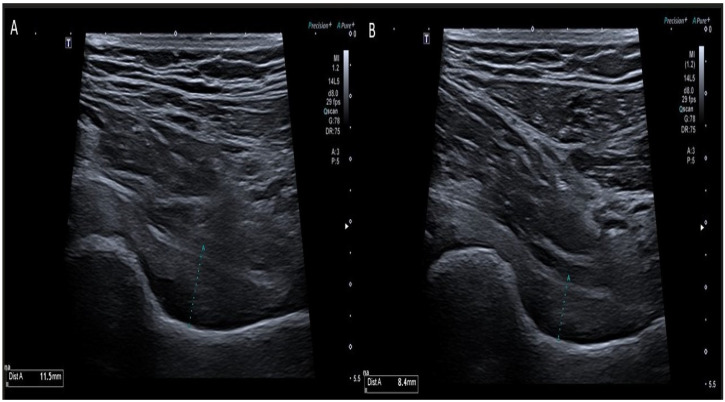
Ultrasound scan of (**A**) the left hip showing progression of the fluid collection to 11.5 mm at the femoral neck and (**B**) the right hip with a fluid collection of 8.4 mm, consistent with non-specific synovitis.

**Figure 3:**
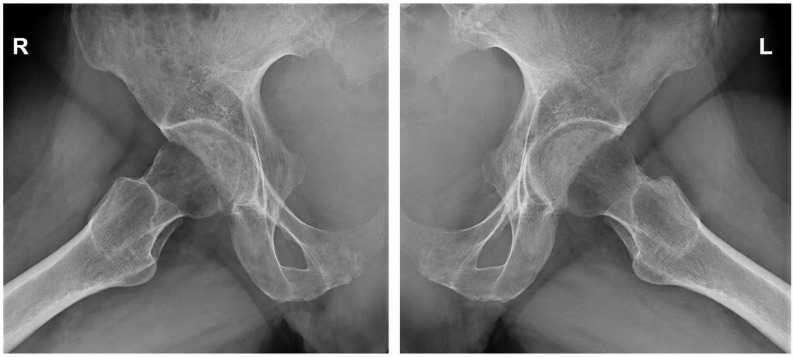
Plain radiographs of the hips, presented separately for each side (**R**) right hip; (**L**) left hip. Both show diffuse osteopenia of the femoral heads without other abnormal findings.

**Figure 4:**
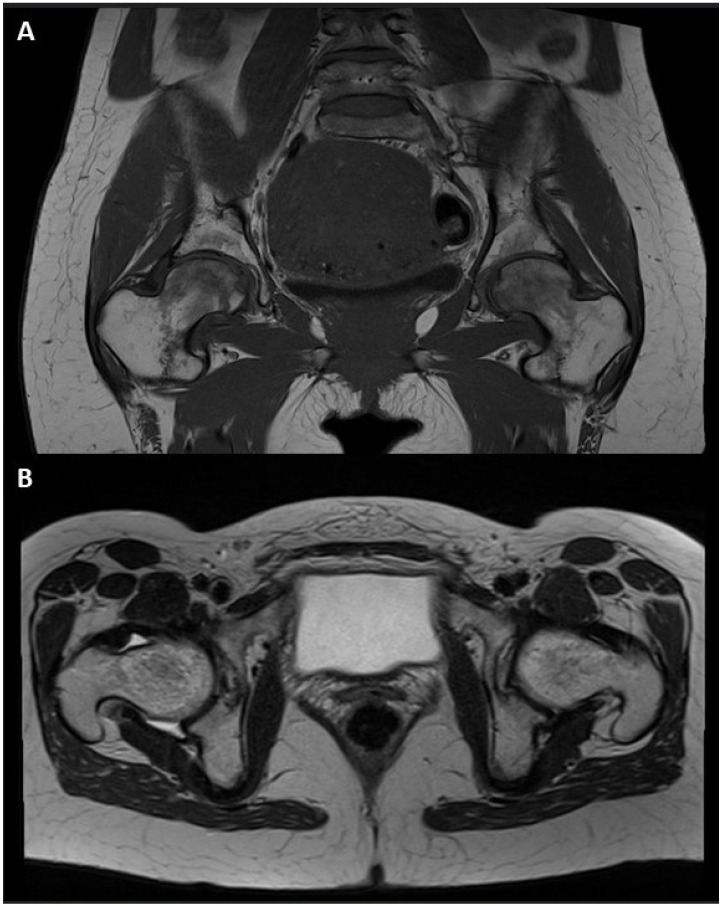
MRI of the hip joints showing diffuse bone marrow oedema involving the entire left femoral head and most of the right femoral head, with low T1-weighted and high T2-weighted signal. Articular cartilage was preserved, and there were no features of avascular necrosis. (**A**) Coronal scan; (**B**) axial scan.

**Figure 5:**
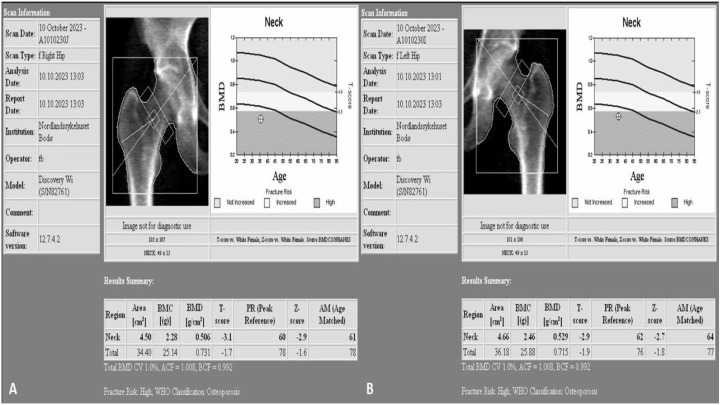
DXA of both hip joints on postpartum day 10. Z-scores: left femoral neck −2.7; right femoral neck −2.9; lumbar spine (L3) −1.3, consistent with a focal reduction of bone mineral density at both femoral necks. (**A**) right hip; (**B**) left hip.

**Figure 6:**
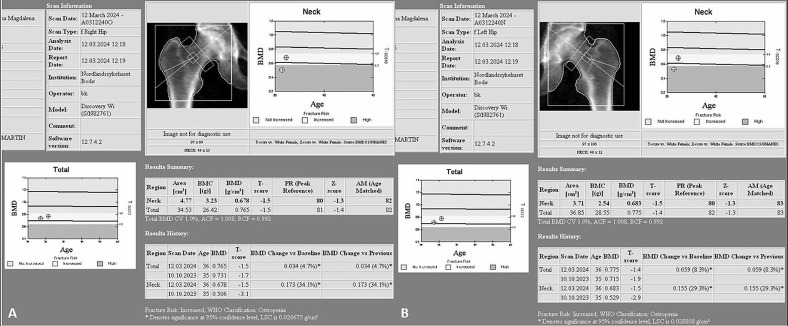
DXA of both hip joints at six months postpartum. Z-scores: left femoral neck −1.3 (Δ +29.3%); right femoral neck −1.3 (Δ +34.1%); lumbar spine essentially unchanged (−1.2 to −1.3). (**A**) right hip; (**B**) left hip.

## Data Availability

The data supporting the findings of this report are held in the electronic patient record system (DIPS) of Nordland Hospital Trust. Because of the sensitive nature of the clinical data, requests for access should be directed to the corresponding author and will be considered subject to applicable data-protection regulations.

## Abbreviations

AVN	Avascular necrosis
AMH	Anti-Müllerian hormone
BMD	Bone mineral density
BMI	Body mass index
CRP	C-reactive protein
CTX	C-terminal telopeptide of type I collagen
DXA	Dual-energy X-ray absorptiometry
IVF-ET	In vitro fertilisation and embryo transfer
LAO	Lactation-associated osteoporosis
MRI	Magnetic resonance imaging
NSAIDs	Non-steroidal anti-inflammatory drugs
PAO	Pregnancy-associated osteoporosis
P1NP	Procollagen type I N-terminal propeptide
PR-TOH	Pregnancy-related transient osteoporosis of the hip
TOH	Transient osteoporosis of the hip
wg	Weeks of gestation
